# M2 macrophages or IL-33 treatment attenuate ongoing *Mycobacterium tuberculosis* infection

**DOI:** 10.1038/srep41240

**Published:** 2017-01-27

**Authors:** A. R. Piñeros, L. W. Campos, D. M. Fonseca, T. B. Bertolini, A. F. Gembre, R. Q. Prado, J. C. Alves-Filho, S. G. Ramos, M. Russo, V. L. D. Bonato

**Affiliations:** 1 Department of Biochemistry and Immunology, Medical School of Ribeirão Preto, University of São Paulo, Ribeirão Preto, São Paulo, Brazil; 2 Department of Pathology, Medical School of Ribeirão Preto, University of São Paulo, Ribeirão Preto, São Paulo, Brazil; 3 Department of Immunology, Institute of Biomedical Sciences, University of São Paulo, São Paulo, Brazil

## Abstract

The protective effects of mycobacterial infections on lung allergy are well documented. However, the inverse relationship between tuberculosis and type 2 immunity is still elusive. Although type 1 immunity is essential to protection against *Mycobacterium tuberculosis* it might be also detrimental to the host due to the induction of extensive tissue damage. Here, we determined whether lung type 2 immunity induced by allergen sensitization and challenge could affect the outcome of *M. tuberculosis* infection. We used two different protocols in which sensitization and allergen challenge were performed before or after *M. tuberculosis* infection. We found an increased resistance to *M. tuberculosis* only when allergen exposure was given after, but not before infection. Infected mice exposed to allergen exhibited lower bacterial load and cellular infiltrates in the lungs. Enhanced resistance to infection after allergen challenge was associated with increased gene expression of alternatively activated macrophages (M2 macrophages) and IL-33 levels. Accordingly, either adoptive transfer of M2 macrophages or systemic IL-33 treatment was effective in attenuating *M. tuberculosis* infection. Notably, the enhanced resistance induced by allergen exposure was dependent on IL-33 receptor ST2. Our work indicates that IL-33 might be an alternative therapeutic treatment for severe tuberculosis.

Tuberculosis (TB), a disease caused by *Mycobacterium tuberculosis*, remains a major public health problem with high morbidity and prevalence. It has killed almost 2 million individuals worldwide, and it is estimated that one-third of the world’s population is infected^1^. Currently, one reason for its high morbidity is the appearance of multidrug- and extensively drug-resistant *M. tuberculosis* strains^2^,^3^. The protective cellular immune response is associated with IFN-γ-producing T cells and classically activated macrophages (M1 macrophages). These cells produce several antibacterial effector molecules, including cytokines, chemokines and NO, which are responsible for the recruitment of inflammatory mononuclear cells, granuloma formation and mycobactericidal activity^4^,^5^. Although IFN-γ is crucial for TB protection and a gold standard parameter of potential TB vaccines, it is apparent that host interaction with *M. tuberculosis* is extremely complex and cannot rely entirely on the induction of IFN-γ-mediated immune responses^6^,^7^,^8^. For instance, Th1 cells and M1 macrophages are associated with type IV hypersensitivity, a pathological immune response associated with necrosis, progressive lung damage, discharge of bacilli from the cavity and enhanced bacterial transmission^9^. Indeed, the paradoxical association between enhanced transmission and polarized inflammation is illustrated by clinical observations that HIV-AIDS individuals who have impaired type 1 immunity and are co-infected with *M. tuberculosis* experience very high bacterial loads but transmit relatively fewer bacteria from person to person^10^,^11^. Collectively, these studies highlight the complex balance between type 1 immunity, tissue damage and bacterial control.

The concept that type 1 and type 2 immunity are mutually inhibitory has been applied in experimental asthma with promising results, as shown by several reports indicating that mycobacterial infections or antigens induce both prophylactic and therapeutic protective effects in allergic lung disease^12^,^13^,^14^,^15^,^16^,^17^,^18^. However, the reverse situation is more complex to ascertain because one must consider bacterial multiplication in addition to preventing tissue immunopathology. Nevertheless, type 2 immunity is associated with M2 macrophages that promote wound repair and tissue regeneration that in turn, might protect lung from tissue destruction^19^. In contrast, M2 macrophages exhibit low antimicrobial immunity, which might be detrimental to *M. tuberculosis* infection^20^,^21^. Here, we determined the impact of lung type 2 immunity induced by ovalbumin as an allergen on the outcome of *M. tuberculosis* infection. For this purpose, we established two basic protocols in which allergen sensitization and challenge was given before or after *M. tuberculosis* infection. We found that increased resistance to *M. tuberculosis* infection was achieved when allergen was administered after infection. Enhanced resistance of allergen-exposed mice compared to infected mice was associated with augmented population of lung CD11c^+^ myeloid cells expressing the mannose receptor (CD206) and increased gene expression of M2 macrophages. Moreover, adoptive transfer of M2 macrophages or systemic IL-33 treatment was also effective in controlling *M. tuberculosis* infection. Importantly, the enhanced resistance induced by the allergen was dependent on the IL-33 receptor ST2. Our data indicate a new mechanism for enhanced resistance to *M. tuberculosis* infection mediated by IL-33/ST2 axis and suggests an alternative therapeutic treatment for severe TB.

## Results

### Allergen exposure after ongoing *M. tuberculosis* infection increases resistance

**Figure 1:**
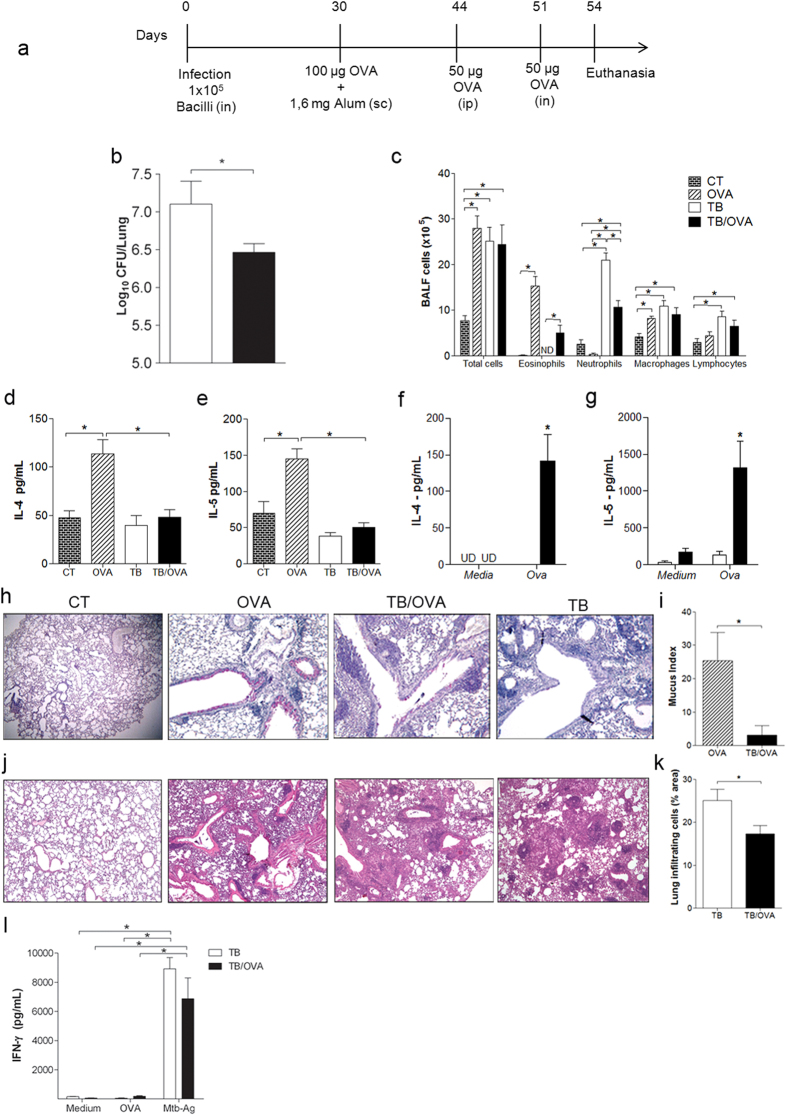
Allergen exposure after ongoing *M. tuberculosis* infection increases resistance. BALB/c mice infected with *M. tuberculosis* were subsequently sensitized and challenged with OVA (**a**). CFU assay (**b**). The data represent the mean ± s.e.m. from one representative (n = 5) of five separate experiments with similar results. Total and differential BALF cell counts (**c**). The data represent the mean ± s.e.m. from 2 independent experiments (n = 6–11). IL-4 and IL-5 production were quantified in BALF (**d**,**e**) or in supernatants of lung cell cultures re-stimulated with OVA (**f**,**g**) were measured by ELISA. The data represent the mean ± s.e.m. from 2 independent experiments (n = 5–10). Representative periodic acid Schiff staining (**h**) and mucus index of lung sections (**i**). Haematoxylin and eosin staining of lung sections, magnification 400x (**j**), and score of lung inflammation (**k**). IFN-γ levels in the supernatants of lung cell culture stimulated with OVA, Mtb-Ag or left non-stimulated (**l**). The data represent the mean ± s.e.m. from 2 independent experiments (n = 4–11). *p < 0.05. CT = non-allergic, uninfected mice; OVA = only-allergy mice; TB/OVA = infected allergic mice; TB = only-infection mice. sc = subcutaneous; ip = intraperitoneal; in = intranasal.

To assess the impact of Th2 immunity on the outcome of mycobacterial infection, we first infected mice with *M. tuberculosis* and then sensitized and challenged with ovalbumin (TB/OVA group) as depicted in Fig. 1a. We expected that by inducing OVA-specific Th2 immunity, the resistance to mycobacterial infection would be impaired. Surprisingly, we found that after allergen challenge, mice were more resistant to *M. tuberculosis* infection as revealed by significantly decreased CFU (Colony Forming Unit) counts in TB/OVA group compared to TB group (Fig. 1b). BALF differential cell counts showed that the number of eosinophils was increased and neutrophils was significantly reduced in TB/OVA group compared with TB group, while there was no difference in macrophage and lymphocyte counts between both groups (Fig. 1c). *M. tuberculosis* infection inhibited OVA-induced lung allergic responses such as eosinophilia, IL-4 and IL-5 production (Fig. 1c–e). However, when lung cell cultures were re-stimulated with Ovalbumin (Ova) the TB/OVA group produced significant levels of IL-4 and IL-5 (Fig. 1f,g). Mucus production revealed by PAS staining (Mucus index) was also reduced in TB/OVA group when compared to OVA group. (Fig. 1h,i). Histopathological analysis showed that the lungs of both groups exhibited a multifocal granulomatous response, but areas of lung infiltrates were significantly decreased in the TB/OVA group compared to the TB group (Fig. 1j–k). Remarkably, IFN-γ production by lung cells cultured with *M. tuberculosis* antigens was not significantly different between the TB and TB/OVA groups (Fig. 1l). Allergen exposure was without effect on the levels of IgG1 or IgG2a against mycobacterial antigens (Supplementary Figure 1a,b). Finally, when the TB/OVA protocol was extended to 80 days post-infection as depicted in Supplementary Figure 2a, we still observed significantly decreased lung CFU counts in the TB/OVA group compared with the TB group (Supplementary Figure 2b). We conclude that airway allergen exposure given after *M. tuberculosis* infection improves mycobacterial clearance and lung pathology. However, the reverse situation as depicted in Supplementary Figure 2c was detrimental to *M. tuberculosis* infection (Supplementary Figure 2d), but reduced Th2-associated allergic responses (Supplementary Figure 2e–h).

Collectively, our results show that Th2 immunity established after, but not before mycobacterial infection, increases resistance to infection, while mycobacterial infection, irrespective of the time, protects against asthma development.

### Allergen exposure after ongoing *M. tuberculosis* infection results in mixed expression of M1/M2 macrophage markers in lungs

**Figure 2:**
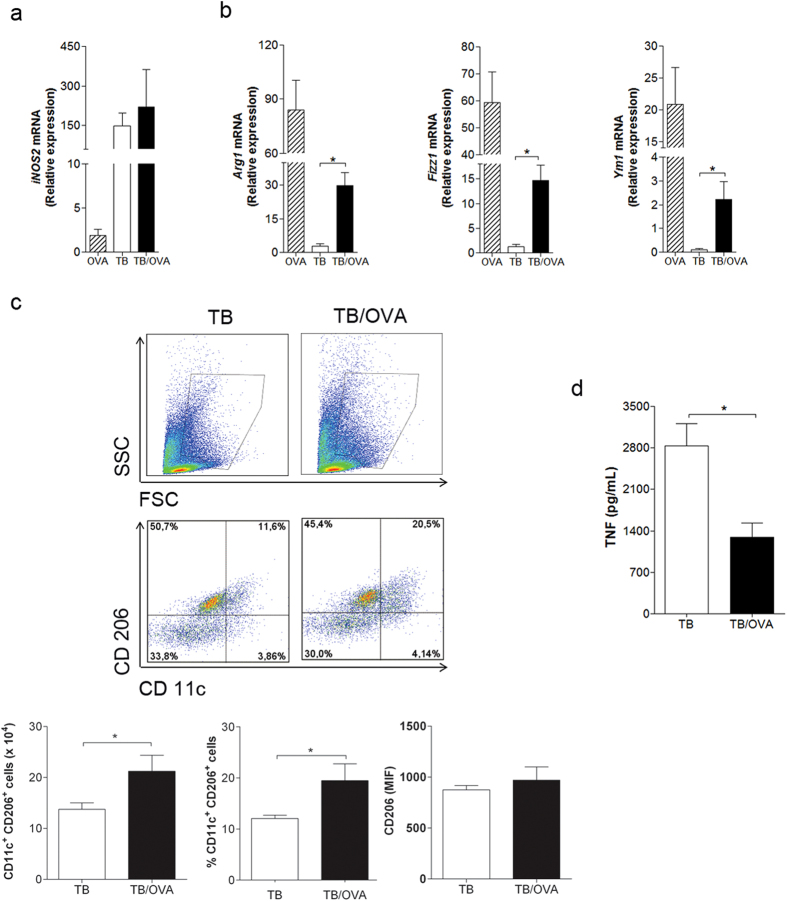
Allergen exposure after ongoing *M. tuberculosis* infection results in the mixed expression of M1/M2 markers in lung. BALB/c mice were infected with *M. tuberculosis* and then sensitized and challenged with OVA, as depicted in Fig. 1a. mRNA expression was measured by quantitative real-time PCR (**a**,**b**). The data represent the mean ± s.e.m. from 3 independent experiments (n = 4–9). Freshly cells from the lung were stained with the combination of surface markers, CD11c and CD206 and examined for flow cytometry. FSC^hi^ SSC^hi^ gate were used to detect CD11c and CD206 expression on cells (**c**). The data represent the mean ± s.e.m. (n = 6–7). TNF levels were evaluated in the lung homogenates (**d**). The data represent the mean ± s.e.m. from 2 independent experiments (n = 14–15) *p < 0.05.

*Mycobacterium tuberculosis* primarily infects macrophages that in turn, when properly activated, express inducible nitric oxide synthase (iNOS) that is associated with resistance to experimental tuberculosis^22^. Because *iNOS* expression is a marker of M1 macrophages^23^ and Th2 immunity induce M2 macrophages, we decided to investigate the expression of M1 and M2 markers in the lung. For this purpose, we evaluated *iNOS* expression as a M1 marker and Arg1, Fizz1 and Ym1 gene expression as M2 markers. Allergen exposure did not affect the expression of *iNOS* as revealed by similar *iNOS* gene expression in the TB group compared to TB/OVA group (Fig. 2a). However, the expression of M2 markers was significantly increased in TB/OVA mice when compared to TB group (Fig. 2b). These results indicate that allergen exposure in infected mice up regulated gene expression associated with M2 macrophages. Because PGE_2_ mediates M2 polarization and affects *M. tuberculosis* growth^24^,^25^, we determined PGE_2_ production in TB/OVA mice. We found that allergen exposure did not affect PGE_2_ production (data not shown). To further characterize the M2-like phenotype in the lungs, we performed FACS analysis on lung cells expressing the mannose receptor (CD206), another M2 marker^26^, and CD11c, which is known to be expressed by lung macrophages and dendritic cells^27^,^28^. We found that the number and the frequency of CD11c^+^CD206^+^ cells were significantly higher in the lungs of TB/OVA mice than in the TB group (Fig. 2c). Finally, we measured the TNF expression as a marker of lung inflammation and found a lower expression of TNF in TB/OVA than in TB group (Fig. 2d). We conclude that allergen exposure increased M2-type myeloid cells and decreased TNF expression, but did not affect *iNOS* expression. Therefore, our results indicate that a mixed M1/M2 lung immunity and lower TNF expression is associated with better outcome of mycobacterial infection.

### Airway adoptive transfer of M2 macrophages enhances resistance to *M. tuberculosis* infection

**Figure 3:**
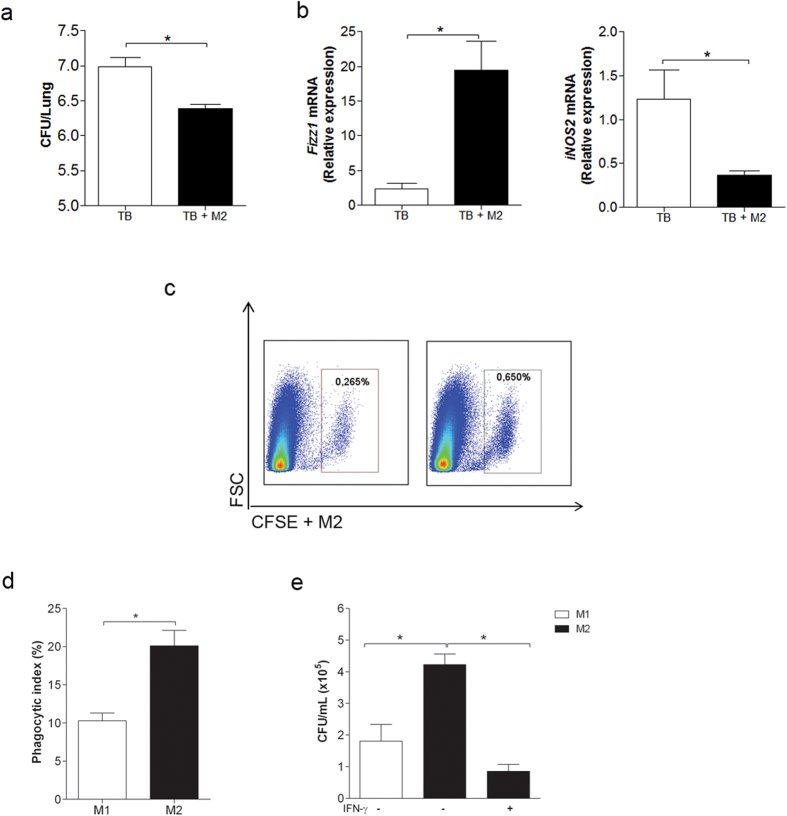
Airway adoptive transfer of M2 macrophages increases the resistance to *M. tuberculosis* infection. Thirty days after *M. tuberculosis* infection, mice underwent M2 cell transfer (2 × 10^6^ cells) by the intra-tracheal route. The CFU count (**a**) and *Fizz1* and *iNOS* gene expression (**b**) were evaluated 8 days post-cell transfer. The data represent the mean ± s.e.m. from 2 independent experiments (n = 10–18). CFSE^+^ M2 macrophages evaluated in the lungs by flow cytometry 24 hours post-cell transfer (**c**). BMDMs were differentiated into M1 or M2 macrophages. The M1 and M2 macrophages were infected with *M. tuberculosis (*MOI 1), and after 4 hours, phagocytic activity was evaluated (**d**). Infected M2 macrophages were treated (+) or not (−) with IFN-γ (200 ng/mL). Untreated infected M1 macrophages (−) were used as controls. After 5 days, the cells were lysed, and serial dilutions were plated for a CFU assay (**e**). The data represent the mean ± s.e.m. from 2 independent experiments (n = 4–6) *p < 0.05.

To directly evaluate the role of M2 macrophages in resistance to *M. tuberculosis* infection, we generated M2 macrophages from bone marrow cultures and administered them to *M. tuberculosis*-infected mice by intra-tracheal route. Remarkably, animals that received M2 macrophages on 30 days post-infection exhibited significantly lower lung CFU counts than infected animals (Fig. 3a). Accordingly, lungs from infected and adoptively transferred mice exhibited higher *Fizz1* but lower *iNOS* gene expression than infected mice (Fig. 3b). Figure 3c confirms that bone marrow-derived M2 macrophages stained with carboxyfluorescein succinimidyl ester (CFSE) reached the lungs. These results indicate that M2 macrophages have a beneficial effect on mycobacterial infection. To mimic the microenvironment occurring *in vivo* in our experiments, we evaluated *in vitro* the phagocytic and microbicidal activity of M2 macrophages in the presence of IFN-γ. In absence of IFN-γ, M2 macrophages showed higher phagocytic activity than M1 macrophages (Fig. 3d) and as expected, M1 macrophages exhibited higher microbicidal activity than M2 macrophages (Fig. 3e). However, upon stimulation with IFN-γ, M2 macrophages increased their bactericidal activity, which was even higher than that obtained with M1 macrophages. Taken together, these results indicate that M2 macrophages in the presence of IFN-γ, a likely situation that occurs *in vivo*, exhibit strong mycobactericidal activity.

### Enhanced resistance to *M. tuberculosis* infection after airway allergen challenge is critically dependent on IL-33 receptor ST2

**Figure 4:**
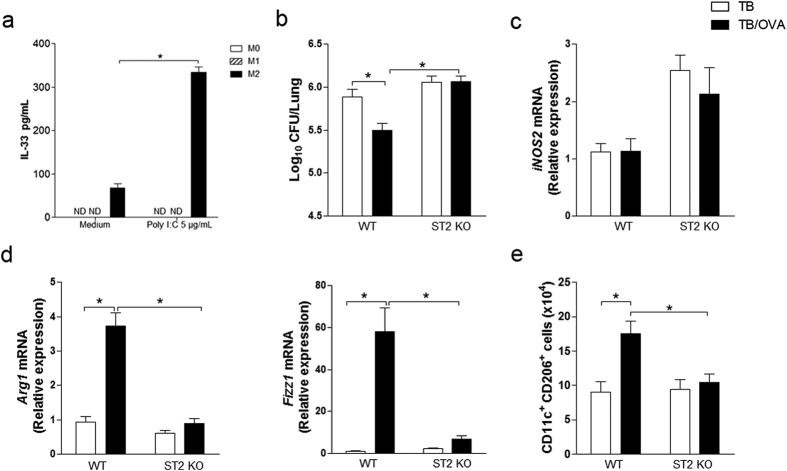
Enhanced resistance to *M. tuberculosis* infection after airway allergen challenge is critically dependent on IL-33 receptor ST2. BMDMs were differentiated into M0, M1 or M2 macrophages and stimulated with Poly IC (5 μg/mL). After 24 hours, IL-33 production was evaluated (**a**). The data represent the mean ± s.e.m. from 1 independent experiment (n = 3) *p < 0.05. BALB/c and ST2KO mice were infected with *M. tuberculosis* and then sensitized and challenged with OVA, as depicted in Fig. 1a. The CFU number (**b**), gene expression (**c**,**d**) and the CD11c^+^CD206^+^ cell population (**e**) were evaluated in the lungs of only-infection WT or ST2KO mice and in the lungs of infected WT or ST2KO mice exposed to allergen. The data represent the mean ± s.e.m. from 2 independent experiments (n = 6–13). *p < 0.05.

Since enhanced resistance to ongoing *M. tuberculosis* infection after allergen exposure was associated with M2-like cells and M2 macrophage cell transfers proved to be beneficial on mycobacterial infection, we were interested in determining the cytokines that could be involved in this process. Among type 2 cytokines, IL-33 plays a pivotal role in type 2 immunity, induces M2 macrophage polarization and it is known to induce pleiotropic and protective functions in Th1 pathologies^29^,^30^,^31^,^32^. Therefore, we first performed *in vitro* experiments with bone marrow differentiated M0, M1 and M2 macrophages stimulated or not with Poly I:C^33^ and found that IL-33 was secreted only by M2 macrophages (Poly I:C stimulated 354 ± 37 vs Medium 68 ± 9.3) (Fig. 4a). To ascertain the role of IL-33 in our model, we first used the IL-33 receptor ST2 knockout mice (ST2KO mice). For this, we evaluated the bacterial loads in the lungs of animals with ongoing mycobacterial infection that were sensitized and challenged with allergen. Infected WT and ST2KO mice (TB groups) showed similar bacterial loads (Fig. 4b). However, after allergen exposure (TB/OVA groups), bacterial load decreased in WT but not in ST2KO mice (Fig. 4b). The *iNOS* gene expression in the lungs of ST2KO-TB and ST2KO-TB/OVA mice was similar, and this expression was higher than those in their respective WT counterparts (Fig. 4c). Accordingly, M2-type phenotype revealed by Arg1 and Fizz1 gene expression as well as by the number of CD11c^+^CD206^+^ cells in the lungs, was significantly lower in ST2KO-TB/OVA mice than in WT-TB/OVA mice (Fig. 4d,e). These results clearly indicate that IL-33 receptor ST2 is critical to induce enhanced resistance to *M. tuberculosis* infection after allergen exposure and gene expression associated with M2 macrophages.

### IL-33 treatment ameliorates ongoing *M. tuberculosis* infection

**Figure 5:**
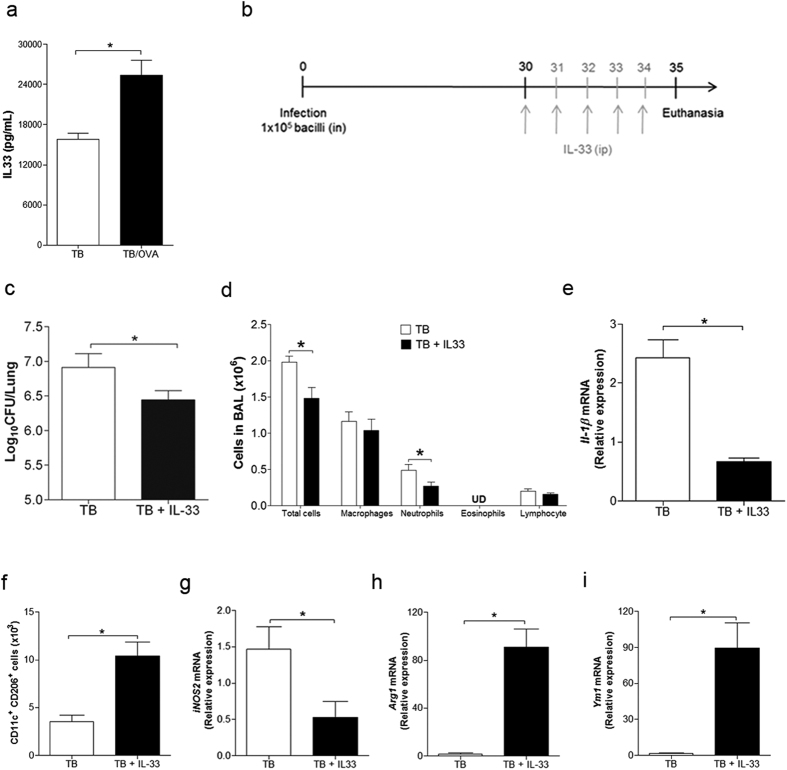
IL-33 treatment ameliorates ongoing *M. tuberculosis* infection. IL-33 levels were evaluated in the lung homogenates of TB/OVA and TB mice (**a**). The data represent the mean ± s.e.m. from 3 independent experiments (n = 15). Thirty days after *M. tuberculosis* infection, mice were treated with recombinant IL-33 (**b**). The CFU number (**c**), BALF total and differential cell counts (**d**), gene expression of *Il-1β* (**e**), and the CD11c^+^CD206^+^ cell population (**f**), *iNOS2* (**g**), *Arg1* (**h**) and *Ym1* (**i**) were evaluated in the lungs of only-infection mice (TB) and the lungs of IL-33-treated infected mice (TB + IL-33). The data represent the mean ± s.e.m. (n = 5–6). *p < 0.05.

Our results indicated that IL-33 production appears to be critical in the protective effect on mycobacterial infection. Therefore, we determined the levels of IL-33 in our model and found that the levels of IL-33 in the lungs of TB/OVA mice, but not in OVA/TB (data not shown), were higher compared to the TB group (Fig. 5a). Hence, to directly test the role of IL-33 in mycobacterial infection, we treated 30-day-infected mice with recombinant IL-33 (Fig. 5b). We found that treatment with IL-33 (TB + IL-33) resulted in a significant decrease in lung CFU counts compared with non-treated infected mice (TB) (Fig. 5c). Since, previous reports indicated that IL-33 treatment induce type 2 cytokines production and recruitment of eosinophils and mononuclear cells^34^,^35^, we determined the magnitude of airway inflammation after IL-33 treatment. We found that IL-33-treated animals showed lower number of total cells and neutrophils in BALF when compared to non-treated TB group and surprisingly IL-33 did not induce eosinophilia (Fig. 5d). These results indicated that IL-33 treatment in *M. tuberculosis*-infected mice has an anti-inflammatory activity. Accordingly, the expression of IL-1β, another broad marker of inflammation, was reduced in IL-33-treated mice (Fig. 5e). Importantly, the number of CD11c-positive cells expressing CD206 increased after IL-33 treatment (Fig. 5f) indicating that IL-33 induced M2-type cells. Accordingly, the lungs of IL-33-treated mice showed decreased expression of M1 marker (*iNOS2*) and increased expression of M2 markers (*Arg1* and *Ym1*) compared to the TB group (Fig. 5g–i).

All together, these results indicate that IL-33 treatment ameliorates ongoing *M. tuberculosis* infection that is associated with increased M2 gene expression and with a population of myeloid cells expressing CD206.

## Discussion

It is now recognized that new therapeutic strategies are needed to improve the control of all forms of *M. tuberculosis* infection^36^. Macrophages are key cells in mycobacterial infection, and although M1 macrophages are essential in pathogen killing, they are also involved in tissue damage^4^,^5^. Therefore, one potential target for therapeutic intervention is the control of lung tissue damage as consequence of the host inflammatory responses. In autoimmune diseases, the control of tissue inflammation is a therapeutic gold standard. However, in infectious diseases, the control of tissue inflammation might lead to uncontrolled pathogen multiplication. M2 macrophages are involved in tissue repair and are associated with Th2 immunity^37^, while mycobacterial infections are known to protect against allergic lung inflammation^12^,^13^,^14^,^17^. Here we asked whether allergen challenge in animals sensitized to OVA after established mycobacterial infection could affect the outcome of ongoing *M. tuberculosis* infection. The findings reported here show that airway allergen challenge in mice previously infected with *M. tuberculosis* (TB/OVA) decreased bacillus multiplication in the lungs. The enhanced resistance experienced by TB/OVA group was associated with gene expression of M2 macrophages, mannose receptor (CD206)-expressing myeloid cells and IL-33. Importantly, airway allergen challenge did not reduce the production of IFN-γ and iNOS2 gene expression that are associated with a protective cellular immune response against experimental TB^6^,^38^,^39^. However, TNF-α production, which is also associated with M1 macrophages, was decreased in our TB/OVA model. This decrease in TNF-α levels might indicate a decreased lung inflammatory response resulting from reduced bacterial load, while *iNOS2* expression might be associated with IFN-γ production. Recently, humoral immunity has been implicated in the control of mycobacterial infection^40^,^41^. However, in our model, allergen challenge did not affect the production of IgG1 and IgG2a against mycobacterial antigens. Neutrophils might have a protective role during *M. tuberculosis* infection^42^,^43^,^44^, however we found a reduction in the number of neutrophils in the BALF and a reduction of mycobacterial load in TB/OVA compared to TB group. Therefore, we favor the notion that the reduced neutrophil infiltration in our model might be a consequence of reduced lung inflammation.

Although CD206 is a marker of M2 macrophages^26^, its expression by myeloid cell population found in the lungs does not necessarily indicate that in fact M2 macrophages are responsible for the beneficial effect on the infection. To directly address the participation of M2 macrophages in resistance to *M. tuberculosis* infection, we performed experiments with M2 macrophages derived from bone marrow-cultures. We found that adoptively transferred M2 macrophages enhanced the resistance of infected mice compared to those animals that did not receive cell transfer. In addition, upon stimulation with IFN-γ, a situation that mimics the *in vivo* microenvironment in the lungs of infected mice, M2 macrophages increased their bactericidal activity. These unexpected findings suggest a new role for M2 macrophages in infections by showing that in addition to controlling tissue damage M2 macrophages might also control bacterial growth in a pro-inflammatory microenvironment. This type of response is similar to the concept of tolerance to infection that is defined as a host defense strategy that reduces the negative impact of infection on host fitness^45^, avoiding tissue damage. In this scenario, the granuloma is initially associated with host protection because it circumvents the infectious focus and avoids spreading. However, in the chronic phase, large granuloma may contribute to the progression of bacillus growth because it dampens the interactions between T lymphocytes and infected cells^9^,^46^. Moreover, the recruitment of highly polarized M1 macrophages may result in tissue necrosis and the dissemination of bacilli. In our study we observed a reduction in the sizes of granuloma-like structures in the lungs of TB/OVA mice. Accordingly, Hammarén *et al*. recently showed that low bacterial loads found in zebrafish infected with *M. marinum* were associated with induction of a type 2 immune response 30 days post-infection^47^. Conversely, the lack of a Th2 immune response was associated with TB progression due to excessive Th1 responses^47^. Excessive inflammation was also shown to occur in *Tir8-*deficient mice that succumb rapidly to infection with *M. tuberculosis,* despite efficiently controlling the infection in different organs^48^. Because Tir8 is a negative regulator of TLR/IL-1R signaling, the high mortality of *Tir8-*deficient mice was attributed to a massive inflammatory response. IL-33/ST2 axis has also been implicated in down modulation of TLR signaling^49^. ST2 is an inhibitor of interleukin 1 receptor and Toll-like receptor 4 signaling and maintains endotoxin tolerance^49^. We found that among type 2 cytokines involved in allergic responses or lung damage, IL-33 was increased in the lungs of infected mice exposed to the allergen. Therefore we studied ST2KO mice in our model. Our results showed that infected ST2KO mice exhibited no difference in lung CFU recovery compared with infected WT mice and are in line with those reported by Wieland *et al*.^50^. Notably, we found that IL-33 receptor ST2 deficiency abolished the enhanced resistance of infected mice exposed to allergen challenge and all type 2 responses associated with enhanced resistance. These findings indicate a pivotal role of IL-33/ST2 axis in protection to *M. tuberculosis* infection. Therefore, we treated infected mice with recombinant IL-33 and found that the treatment reproduced the observed resistance to *M. tuberculosis* infection associated with allergen exposure or M2 macrophage cell transfers. Our work are in line with previous work showing a protective effect of IL-33 treatment in cerebral malaria^51^, another example of severe inflammation. Because we found decreased *iNOS2* expression during IL-33 treatment and in experiments with M2 cell transfers, we suggest that other alternative mycobactericidal activity might be operating. Recent report showed that itaconic acid produced by macrophages has antimicrobial activity^52^. Also, the production of itaconic acid is regulated by IRG1 gene, which in turn, is activated by molecules (IL-10, HO-1 and CO) that can be associated with M2 macrophages^53^,^54^. However, IL-33 was described as a promising adjuvant that increased the immunogenicity of a DNA vaccine expressing *M. tuberculosis* antigen 85B^55^, suggesting that it could be an effective tool for the development of vaccines against TB. It should be noted that the beneficial effects of type 2 immunity are time- and context-dependent. For instance, established type 2 immunity before mycobacterial infection is detrimental to the host as previously shown by Potian for helminth infection^21^ and by us in the OVA/TB model (Supplementary Figure 1c,d). In a similar approach employed with a keratitis model induced by *Pseudomonas aeruginosa* corneal infection, Hazlett *et al*. showed that treatment with recombinant IL-33 was protective^56^. It is highly desirable that new therapies for TB should involve two approaches: to improve cellular effector mechanisms and to prevent lung damage^36^. Indeed, tissue damage as a consequence of sustained inflammation causes permanent debility even in cured TB patients^57^. Our results show the potential therapeutic effects of IL-33 in established mycobacterial infection and highlight that IL-33 might represent an alternative therapeutic agent for advanced TB.

## Materials and Methods

### Animals

Female BALB/c and ST2KO mice (6–8 weeks) were obtained from the breeding facility of the Medical School of Ribeirão Preto, University São Paulo (FMRP-USP). The ST2KO mice^58^ were backcrossed to the BALB/c background for 8 generations^49^. All animals were housed in ABSL3 and provided sterile food and water. All animal experiments were conducted in accordance with the ethical guide on the use of animals of FMRP-USP, and were approved by the Ethics Committee on Animal Use of FMRP-USP (protocol number 133/2012).

### *M. tuberculosis* infection

H37Rv *M. tuberculosis* (American Type Culture Collection 27294, Rockville, MD) was grown in Middlebrook 7H9 Broth (Difco Laboratories, Detroit, MI, USA) at 37 °C for 7 days and harvested as previously described^59^. Mice were infected by administration of 1 × 10^5^ bacilli by the intranasal route. The lungs were evaluated at 30 or 80 days of infection.

### Experimental design

#### OVA/TB protocol

Mice were sensitized with 100 μg of OVA (Sigma, St. Louis, MO, USA) and 1.6 mg of aluminum hydroxide adjuvant by the subcutaneous (s.c.) route. Fourteen days after the first sensitization, the mice underwent a second sensitization with 50 μg of OVA in 100 μL of saline by intraperitoneal (i.p.) injection. Seven days after the second sensitization, the mice were challenged with 100 μg of OVA by intranasal administration. The mice were infected with 1 × 10^5^ *M. tuberculosis* bacilli by the intranasal (i.n.) route three days after the first challenge.

#### TB/OVA protocol

Mice were infected with *M. tuberculosis*, followed by sensitization and challenge with OVA, as described. For the extended protocol (80 days of infection), the mice received two more challenges on days 64 and 77.

### Bronchoalveolar lavage

Bronchoalveolar lavage fluid (BALF) was obtained three days after challenge, as described previously^12^,^13^.

### Flow cytometry

Cell suspensions (1 × 10^6^ cells) obtained after lung digestion^60^ were incubated with supernatant of 2.4G2 cell lineage (containing antibodies anti-FcγRII/III) for 40 min at 4 °C. The cells were then incubated for 30 min at 4 °C with monoclonal antibodies purchased from BD Biosciences: CD11c-PECy^TM^ 7 (HL3) and CD206-Alexa Fluor^®^ 488 (MR5D3). The stained cells were washed with 2% FBS (Fetal Bovine Serum) and fixed with PBS containing 1% formaldehyde. The samples (100,000 events) were assessed using a FACSCanto^TM^ II cytometer (BD, San José, CA, USA) and CellQuest software (BD Biosciences). The cells were analyzed using FlowJo software (v 7.6.5, Tree Star, Ashland, OR). Initially, the cells were gated on FSC-A and FSC-H for doublet exclusion, followed by a strategy to gate on FSC (size) and SSC (granularity) to select a population compatible with macrophages, within which the CD11c^+^CD206^+^ population was evaluated.

### Mucus production and lung inflammation

The right upper lung lobes were collected from individual animals, fixed in buffer containing 3.7% of formaldehyde and then embedded in paraffin blocks and sectioned. The lung sections were stained with hematoxylin and eosin (HE) to characterize the cellular infiltrate. Periodic Acid Schiff (PAS) staining was used to evaluate mucus production as previously described^12^. The sections of stained lung were analyzed using a Leitz Aristoplan microscope (Wild Leitz GMBH, Germany) equipped with a camera (Leica Microsystems LTDA, Germany). The images were analyzed using *ImageJ software* (NIH Image, Bethesda, MD, USA). Lung-infiltrating cells were used to determine the ratio between the lung area with infiltrating cells and the total area of photographed lung tissue. Mucus index was obtained for ratio between the area positively stained (PAS-positive) and the bronchial area of photographed lung.

### Cytokines

The levels of cytokines in BALF, lung homogenates and lung cell cultures were measured using ELISA kits obtained from BD Biosciences, BD *OptEIA*^*TM*^ *Set* (San Diego, CA, USA) (IFN-γ, IL-4, IL-5 and TNF) and R&D Systems (Minneapolis, MN, USA) (IL-33). Lung cells were prepared as previously described^60^ and cultured for 48 hours with OVA (100 μg/mL) or mycobacterial antigens (10 μg/mL) obtained from the sonicated mycobacteria^59^. As controls, lung cells were left unstimulated or were stimulated with Con-A (40 μg/mL).

### ELISA for antibody detection

Anti-mycobacterial antibody IgG1 and IgG2a were detected in serum from TB/OVA and TB mice as previously described^61^. It was used biotin-conjugated anti-mouse IgG1 (A85-1) and IgG2a (R19-15) antibodies. The optical density (OD) was measured at 490 nm.

### RT-PCR

The right or left lung lobe was homogenized in 1 mL of TRIzol reagent (Invitrogen) and purified using chloroform. RNA was precipitated with isopropyl alcohol and 70% ethanol. cDNA synthesis was performed using SuperScript^TM^ II Reverse Transcriptase (Invitrogen by Life Technologies^TM^, Carlsbad, CA, USA). Real-time PCR was performed using Maxima SYBR Green/ROX qPCR Master Mix (2x) (Thermo Fisher Scientific, Inc., Waltham, MA, USA) in the StepOnePlus^TM^ Real-Time PCR System (Applied Biosystems, Foster City, CA, USA). The samples were amplified using the following conditions: initial denaturation at 95 °C for 10 minutes, followed by 40 cycles of 95 °C for 15 seconds, an annealing phase at 58 °C for 30 seconds and an extension phase at 72 °C for 30 seconds. The samples were analyzed using the threshold cycle (C_t_). Gene expression was calculated as 2^−(ΔΔCt)^, where ΔΔCt = ΔC_t_ (sample) − ΔC_t_ (calibrator) and where ΔC_t_ (sample) = C_t_ (target gene) − C_t_ (normalizer = *β-Actin*). The following primer sequences were used for detection: *β-Actin* (forward, 5′-CCC TAG GCA CCA GGG TGT GA-3′; reverse, 5′-GCC ATG TTC AAT GGG GTA CTT C-3′), *iNOS* (forward, 5′-TGC TGT TCT CAG CCC AAC AAT A-3′; reverse, 5′-GTC CAG GGA TTC TGG AAC ATT CT-3′), *Arg1* (forward, 5′-CAA AAG GAC AGC CTC GAG GAG-3′; reverse, 5′-CCC GTG GTC TCT CAC GTC AT-3′), *Fizz1* (forward, 5′-CCT GAG ATT CTG CCC CAG GAT-3′; reverse 5′-TTC ACT GGG ACC ATC AGC TGG-3′), and *Ym1* (forward, 5′-TCA CAG GTC TGG CAA TTC TTC TG-3′; reverse, 5′-ACT CCC TTC TAT TGG CCT GTC C-3′).

### Adoptive transfer of M2 macrophages and *in vivo* IL-33 treatment

Thirty days post-infection, mice received 2 × 10^6^ bone marrow-derived M2 macrophages by the intra-tracheal route or were treated with recombinant IL-33 (BioLegend, San Diego, CA) (1 μg/mouse) by the intranasal route (five doses total at 2-day intervals) as previously reported^51^,^62^. A CFU assay was performed at 8 days post-adoptive cell transfer or 24 hours after the last treatment with IL-33.

### *In vitro* infection of macrophages

Bone marrow precursors were incubated with RPMI medium containing 20 ng/mL macrophage colony-stimulating factor (M-CSF) (PeproTech, Rocky Hill, NJ, USA) in petri plates (BD OPTILUX). The culture medium was replaced on day 3, and 5 ng/mL M-CSF was added. After 7 days, 1 × 10^7^ BMDMs were plated under conditions stimulating M1 or M2 macrophage polarization via 200 ng/mL IFN-γ (R&D Systems, Minneapolis, MN, USA) and 1 μg/mL LPS (Sigma-Aldrich, St. Louis, MO, USA) or 30 ng/mL IL-4 (R&D Systems, Minneapolis, MN, USA), 30 ng/mL IL-13 (R&D Systems, Minneapolis, MN, USA) and 30 ng/mL IL-33 (R&D Systems, Minneapolis, MN, USA), respectively, for 48 hours. Then, 2.5 × 10^5^ cells were plated in 24-well plates (Corning^®^, NY, USA), and after 4 hours, the cells were infected with *M. tuberculosis* (MOI 1). The culture medium was replaced with RPMI-1640 containing 10% FBS without antibiotics after 4 hours. The M1 and M2 macrophage cultures were left untreated or were treated with IFN-γ (200 ng/mL). The medium was replaced every 2 days. At the indicated times, the cells were washed and lysed with 0.05% saponin. The cell lysates were serially diluted in PBS, and 100 μL was placed on Middlebrook 7H11 agar. CFU counts were determined after 30 days of incubation at 37 °C in 5% CO_2_.

### Poly I:C stimulated macrophages

BMDMs were differentiated into M0, M1 or M2 macrophages, as previously described. The cells (1 × 10^6^) were cultured in 24 wells plate and stimulated with 5 μg/mL de Poly I:C. After 24 hours, IL-33 production was evaluated by ELISA.

### Statistical analysis

The data were analyzed with StatSoft, Inc. (2004) STATISTICA software version 7 (Tulsa, OK, USA). The statistical significance of differences between groups was estimated using a one-tailed t test for parametric data and a Mann-Whitney U-test for non-parametric data. The data from experiments with three or more groups were analyzed using one-way ANOVA with Tukey’s test for parametric data and the Kruskal-Wallis test for non-parametric data. The data were expressed as the mean ± standard error of mean (s.e.m.). A p*-*value of less than 0.05 was considered significant.

## Additional Information

**How to cite this article**: Piñeros, A. R. *et al*. M2 macrophages or IL-33 treatment attenuate ongoing *Mycobacterium tuberculosis* infection. *Sci. Rep.* **7**, 41240; doi: 10.1038/srep41240 (2017).

**Publisher's note:** Springer Nature remains neutral with regard to jurisdictional claims in published maps and institutional affiliations.

## Supplementary Material

Supplementary Information
