# Supplementary material for: M2 macrophages or IL-33 treatment attenuate ongoing Mycobacterium tuberculosis infection
Source: Sci Rep. 2017 Jan 27;7:41240. doi: 10.1038/srep41240 (PMC5269597; doi:10.1038/srep41240)
Supplement: Supplementary Information [file srep41240-s1.doc]

M2 macrophages or IL-33 treatment attenuate ongoing *Mycobacterium tuberculosis* infection

AR Piñeros, LW Campos, DM Fonseca, TB Bertolini, AF Gembre, RQ Prado, JC Alves-Filho, SG Ramos, M Russo, VLD Bonato


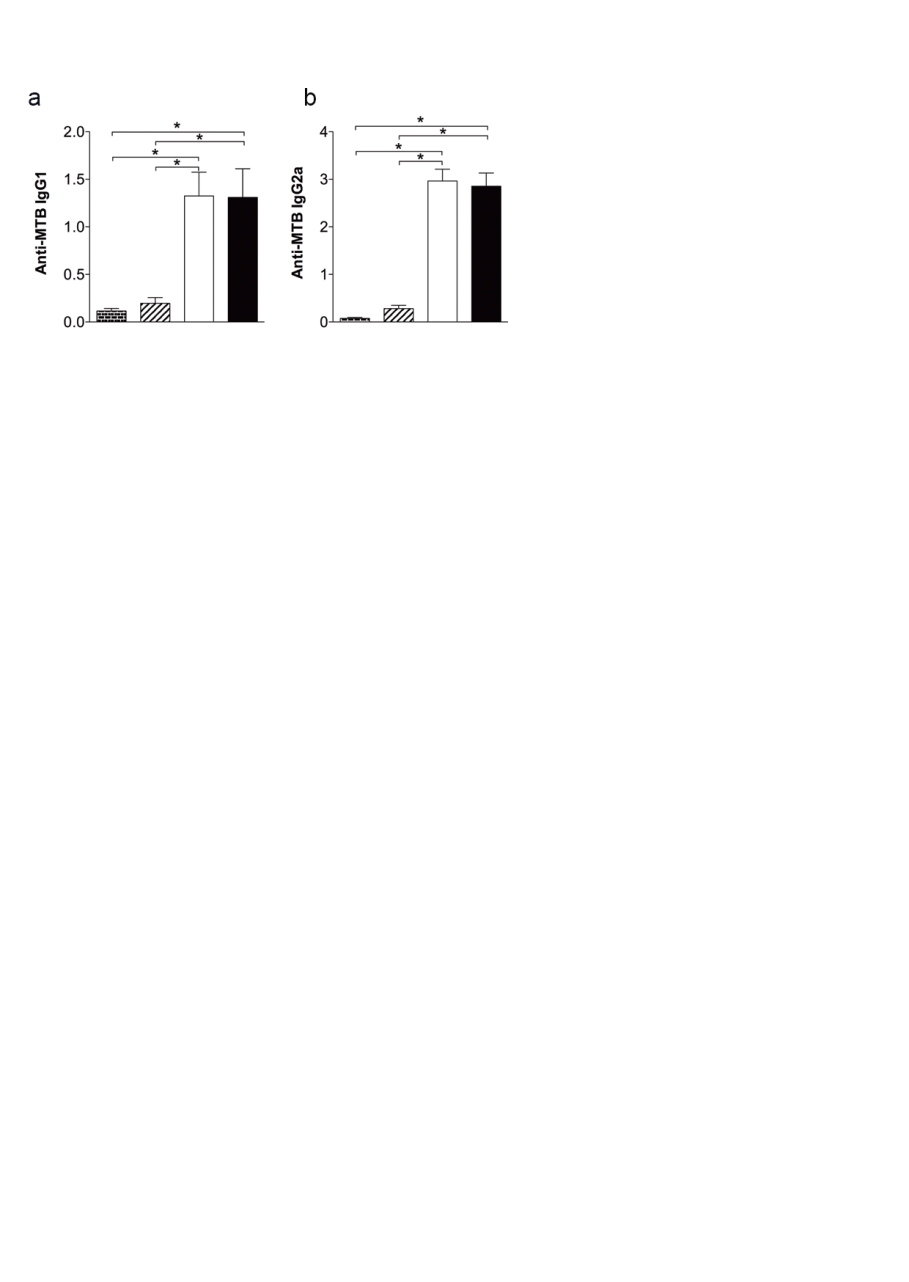


**Supplementary Figure 1. Allergen exposure after ongoing *M. tuberculosis* infection did not affect the production of IgG1 and IgG2a against mycobacterial antigens.** BALB/c mice infected with *M. tuberculosis* were subsequently sensitized and challenged with OVA. IgG1 against mycobacterial antigens (anti-Mtb IgG1) **(a)** and anti-Mtb IgG2a **(b)** were quantified in serum, 72 after challenge with OVA. Data represent the mean ± s.e.m. from 2 independent experiments (n = 9-11). CT = non-allergic, uninfected mice; OVA = only-allergy mice; TB/OVA = infected allergic mice; TB = only-infection mice.


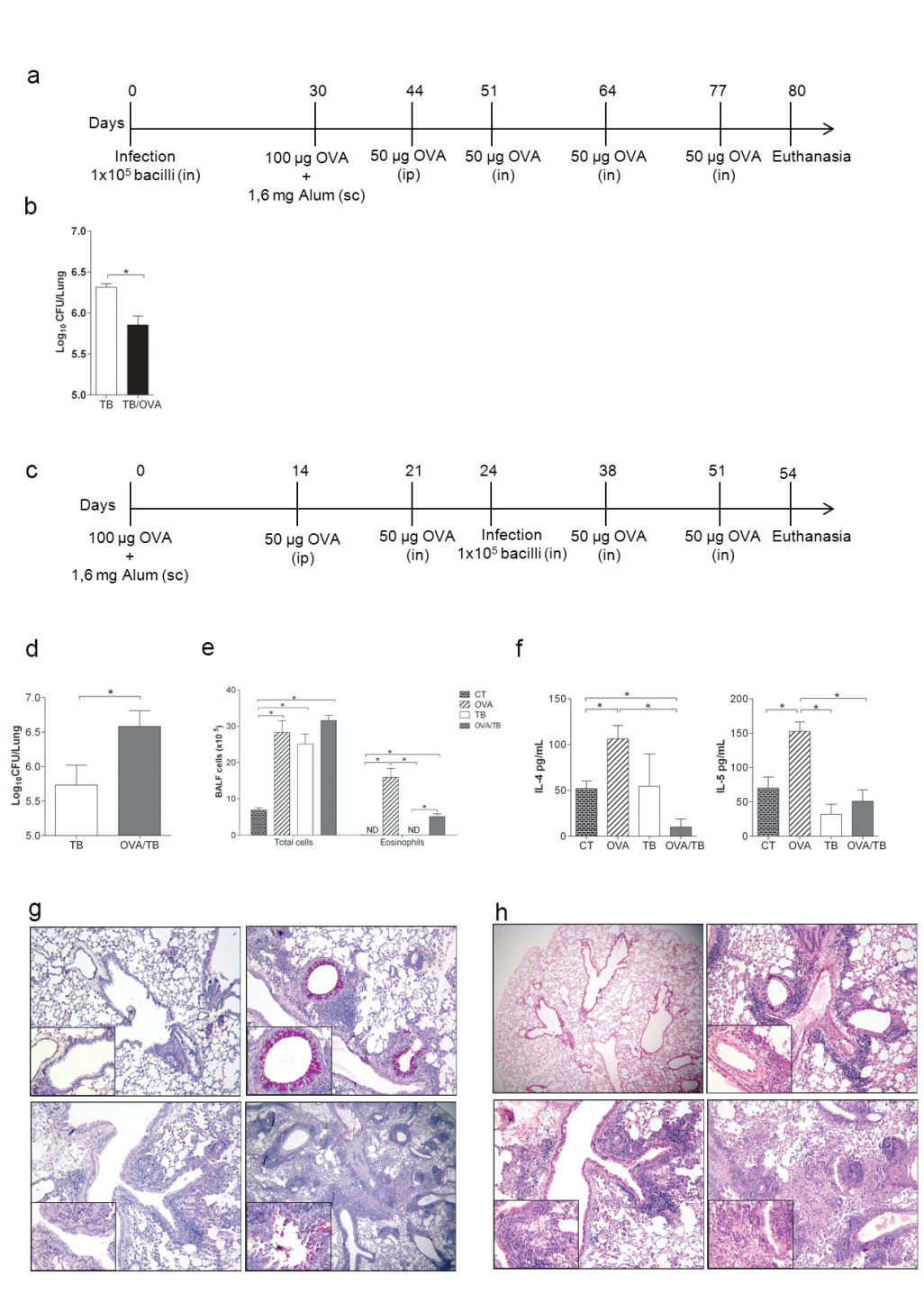


**Supplementary Figure 2. Allergen exposure prior *M. tuberculosis* infection increases the susceptibility**. BALB/c mice were infected with *M. tuberculosis* and then sensitized and challenged with OVA **(a)**. CFU counts were evaluated at 80 days post-infection **(b)**. The data represent the mean ± s.e.m. from 2 independent experiments (n =9-13). BALB/c mice were sensitized and challenged with OVA, followed by intranasal *M. tuberculosis* infection. The mice were subsequently challenged with OVA, and their lungs were evaluated 30 days post-infection **(c)**. CFU counts were evaluated in samples of digested lungs **(d)**. The data represent the mean ± s.e.m. from one representative (n = 5) of three separate experiments with similar results. * p < 0.05. CT = non-allergic, uninfected mice; OVA = only-allergy mice; OVA/TB = allergic infected mice; TB = only-infection mice. BALF total cell counts and eosinophil number **(e)**. The data represent the mean ± s.e.m. from 3 independent experiments (n = 12-20). Cytokine levels in the supernatants of BALF were measured by ELISA **(f)**. The data represent the mean ± s.e.m. from 3 independent experiments (n = 5-18). Representative periodic acid Schiff **(g)** and haematoxylin and eosin **(h)** staining of lung sections, magnification 400x. * p < 0.05. sc = subcutaneous; ip = intraperitoneal; in = intranasal.
